# Comparison of the Clinical Features of Japanese Patients with Primary Biliary Cirrhosis in 1999 and 2004: Utilization of Clinical Data When Patients Applied to Receive Public Financial Aid

**DOI:** 10.2188/jea.17.210

**Published:** 2007-12-19

**Authors:** Fumio Sakauchi, Asae Oura, Hirofumi Ohnishi, Mitsuru Mori

**Affiliations:** 1 Department of Public Health, Sapporo Medical University School of Medicine

**Keywords:** Liver Cirrhosis, Biliary, Public Financial Aid, Clinical Findings, Laboratory Findings, Antimitochondrial Antibodies

## Abstract

**BACKGROUND:**

In Asia there are few reports considering time intervals in the examination of clinical features of primary biliary cirrhosis (PBC). Therefore, we tried to compare the characteristics of patients with PBC in two different years.

**METHODS:**

In two fiscal years (1999 and 2004), 9,761 and 13,142 patients with symptomatic PBC were registered to receive public financial aid from the Ministry of Health, Labour and Welfare of Japan, respectively. For the present study, clinical data from 2,127 patients in 1999 and 6,423 ones in 2004 were available. We compared the data in the two different years, including sex, age, major symptoms, and laboratory data.

**RESULTS:**

Male/female ratios were the same figure (0.13 for 1999 and 2004). The median age was significantly older in 2004 than in 1999 (59 years for 1999, 63 years for 2004, respectively, p<0.01). Jaundice and esophageal varices were found significantly less frequent in 2004 than in 1999 (p<0.01 for each item). Levels of total bilirubin, γ-glutamyl transpeptidase (γ-GTP), total cholesterol, and immunoglobulin M were significantly lower in 2004 than in 1999 (p< 0.02 for total bilirubin, and p<0.01 for other each item). The positive rate of antimitochondrial antibodies was significantly higher in 1999 than in 2004 (87.0% for 1999, 83.5% for 2004, respectively, p<0.01)). Complicated autoimmune dis­eases such as Sjögren's syndrome, rheumatoid arthritis, and chronic thyroiditis were found significantly more frequent in 2004 than in 1999 (p<0.01 for each item).

**CONCLUSIONS:**

Among the patients with PBC in 2004, an increase in median age, and lower levels of laboratory data such as γ-GTP have been found compared to 1999. These results may show an accu­mulation of patients with better prognosis and the recent medical progress in controlling patients with PBC.

Primary biliary cirrhosis (PBC) is a chronic cholestatic disorder characterized by the progressive, nonsuppurative inflammation and destruction of small bile ducts, and the presence of antimitochondrial antibodies (AMA) in the sera. PBC is considered to be associated with disturbances in both cellular and humoral immunity.^1^ There are two known clinical types of PBC, i.e, one is asymptomatic PBC which shows no symptoms of hepatic disorder, and the other is symptomatic PBC which has various clinical symptoms and signs, such as pruritus and jaundice.^1^^,^^2^ In Japan symptomatic PBC was specified as one of "the intractable diseases" from 1990. Patients with symptomatic PBC who want to receive public financial aid for the treatment from the Ministry of Health, Labour and Welfare must sign agreements and write applications. Then they are registered and can receive public financial aid. The recognition of patients with symptomatic PBC is conducted by each prefecture.

Although PBC has been described in virtually all parts of the world,^3^ most of the epidemiologic data have been derived from Europe,^4^^,^^5^ and in Asia, and there are few reports considering time intervals in the examination of clinical features of PBC. Previously our cross-sectional study showed clinical futures of Japanese patients with PBC in 1999.^6^ In the present study, we tried to compare the characteristics of patients with PBC in two different years by utilization of the clinical data when they applied to receive public financial aid.

## METHODS

In the present study, patients whose conditions met one of the criteria below were diagnosed as having PBC following the previous reports in Japan.^7^^,^^8^

1. Chronic non-suppurative destructive cholangitis (CNSDC) is histologically observed, and laboratory data do not contradict PBC.
2. AMA is positive. CNSDC is not histologically observed, but histological findings are compatible with PBC.
3. Histological examination is not performed, but AMA is positive, and clinical findings and course indicate PBC.

For the patients with symptomatic PBC, the following information was collected from the records: sex, date of birth, date of diagnosis, estimated onset time, symptoms and physical findings, complicated autoimmune diseases, laboratory data including serum levels of total bilirubin, alkaline phosphatase (ALP), γ-glutamyl transpeptidase (γ-GTP), total cholesterol (T-Chole), immunoglobulin M (IgM), and AMA.

In fiscal year 1999, 9,761 prevalent cases with symptomatic PBC were registered. We could obtain clinical data of 6,527 patients from the Research Committee of Intractable Hepatic Diseases. From 2001, the Ministry of Health, Labour and Welfare started inputting data of patients with intractable diseases collected from prefectures in Japan, and from 2004 electric devices including those clinical data were available. Before 2002, patients with intractable disease applied for financial aid every three years, but after 2003 they have to apply for it every year. In the fiscal year of 2004, 13,142 prevalent cases with symptomatic PBC were registered, and we were permitted to use the clinical data of 6,423 patients provided from the Ministry of Health, Labour and Welfare. Unfortunately, we could not access all of the data from registered patients with PBC in those two years because several prefectures did not provide the data and there were many blank spaces in data regarding resident areas of the patients in 1999. Therefore, 2,127 cases in 1999 and 6,423 cases in 2004 who lived in the same prefecture (32 prefectures) were used to examine for the present study.

We compared symptoms and physical findings, laboratory data, and complicated autoimmune diseases in 1999 and 2004. In the present study, frequencies of items in the clinical data were analyzed, excluding "unclear" or blank spaces. Statistical analysis was performed using SPSS^®^ version 13 (SPSS Inc.). The chi-square test was used for comparing the proportions of two groups, and the Mann-Whitney test was used to evaluate differences in clinical variables. P < 0.05 was considered significant.

## RESULTS

### Sex and Age

Table 1 presents the demographic characteristics of the patients with PBC examined in this study. The male/female ratios were the same figure (0.13 for 1999 and 2004). The median ages of the patients in 1999 and 2004 were 59 and 63 years, respectively. The median age of the patients with PBC was significantly older in 2004 than in 1999 (p<0.01). The highest frequencies were in the 60s for the two years (33.2% in 1999 and 33.9% in 2004, respectively). The proportion of the groups aged 20-69 years decreased, while the groups aged 70 years or older increased in 2004 compared to 1999.

**Table 1.** Demographic characteristics of the patients with primary biiary cirrhosis in 1999 and 2004.

	1999	2004	P value
Number of subjects	n=2,127	n=6,423	

Male/Female ratio	0.13 (250/1,877)	0.13 (753/5,670)	

Age (median, interquartile range)	59 years (51-67)	63 years (55-70)	<0.01*

Age(year) (%)			
-19	2 (0.1)	8 (0.1)	
20-29	10 (0.5)	33 (0.5)	
30-39	58 (2.7)	128 (2.0)	
40-49	322 (15.1)	555 (8.6)	
50-59	692 (32.5)	1,801 (28.0)	
60-69	707 (33.2)	2,175 (33.9)	
70-79	300 (14.1)	1,476 (23.0)	
80+	36 (1.7)	247 (3.8)	

Total	2,127 (100)	6,423 (100)	

*: Mann-Whitney test for 1999 vs. 2004

### Symptoms and Physical Findings

In 1999, pruritis was present in 55.5%, jaundice in 11.8%, and esophageal varices in 21.4% of the patients. While, in 2004, pruritis was present in 57.5%, jaundice in 7.2%, and esophageal varices in 16.5% (Table 2). Statistical significance was not found in the proportion of pruritis, but jaundice and esophageal varices were found significantly less frequently in 2004 than in 1999 (p<0.01 for each item).

**Table 2.** Prevalence of selected symptoms and physical findings among patients with primary biliary cirrhosis in 1999 and 2004.

	1999	2004	P value*
Pruritus	55.5% (1,154/2,080)	57.5% (3,664/6,371)	0.10
Jaundice	11.8% ( 248/2,094)	7.2% ( 454/6,348)	<0.01
Esophageal varices	21.4% ( 397/1,857)	16.5% (1,002/6,072)	<0.01

*: Chi square test for 1999 vs. 2004

### Laboratory Data

Key laboratory data are summarized in Table 3. Levels of total bilirubin seemed to be almost the same among the patients in 1999 and 2004. We calculated 95 percentiles of the levels of total bilirubin for the two years and found that they were 3.0 mg/dL in 1999 and 2.2mg/dL in 2004, respectively. Regarding levels of ALP, significant difference did not exist between the two years. Whereas levels of γ-GTP, T-Chole, and IgM were significantly lower in 2004 than in 1999 (p<0.01 for each item). The positive rate of AMA was significantly higher among the patients in 1999 than in 2004 (87.0% for 1999, 83.5% for 2004, respectively, p<0.01).

**Table 3.** Laboratory findings of patients with primary biliary cirrhosis in 1999 and 2004.

	1999		2004		P value*
	
	Median	Interquartile range	Median	Interquartile range	
Total Bilirubin (mg/dL)	0.6 (n=2,127)	0.5 - 1.0	0.7 (n=6,248)	0.5 - 0.9	0.02*
ALP (IU/L)	363 (n=2,108)	241 - 569	360 (n=6,317)	263 - 511	0.72*
γ-GTP (IU/L)	87 (n=2,127)	38 - 198	62 (n=6,328)	31 - 133	<0.01*
Total Cholesterol (mg/dL)	202 (n=2,127)	172 - 231	197 (n=5,860)	171 - 223	<0.01*
IgM (mg/dL)	360 (n=1,690)	221 - 571	242 (n=4,096)	157 - 373	<0.01*

AMA positivity	87.0% (1,761/2,023)		83.5% (3,932/4,710)		<0.01**

*: Mann-Whitney test for 1999 vs. 2004

**: Chi square test for 1999 vs. 2004

AMA: antimitochondrial antibody

### Complicated Autoimmune Diseases

Complicated autoimmune diseases such as Sjögren's syndrome, rheumatoid arthritis, and chronic thyroiditis were found significantly more frequently in 2004 than in 1999 (17.0%, 7.3%, and 4.7% in 1999, 20.7%, 10.3%, and 12.4% in 2004, respectively, p<0.01 for each item) (Table 4).

**Table 4.** Prevalence of complicated autoimmune diseases among patients with primary biliary cirrhosis in 1999 and 2004.

Autoimmune diseases	1999	2004	P value*
Sjögren's syndrome	17.0% ( 310/1,827)	20.7% ( 895/4,322 )	<0.01
Rheumatoid arthritis	7.3% ( 146/2,005 )	10.3% ( 395/3,822 )	<0.01
Chronic thyroiditis	4.7% ( 99/2,127 )	12.4% ( 487/3,914 )	<0.01

*: Chi square test for 1999 vs. 2004

## DISCUSSION

In Western countries, it has been reported that 90% to 95% of patients with PBC are women, with the median age at the time of diagnosis in the early 50s.^9^^-^^11^ In Japan, the Research Committee on the Epidemiology of Intractable Diseases conducted two rounds of nationwide surveys of PBC in 1992 and 1997.^12^^,^^13^ These surveys reported that the male/female ratio was 0.11 in 1992 and 0.12 in 1997, respectively. The male/females ratios in the present study were 0.13 in 1999 and 2004 so that these ratios were considered to be in approximate agreement with the two reports from the previous nationwide surveys in Japan. The median age of the patients with PBC was significantly older in 2004 than in 1999 (59 years in 1999 and 63 years in 2004, respectively). The main reason regarding this increase of median age was that the proportion of the groups aged 20-69 years decreased but in contrast the groups aged 70 years or older increased in 2004 compared to 1999. One of the explanations of the increase of median age may owe to an accumulation of patients with better prognosis and the recent medical progress in controlling patients with PBC.

Jaundice and esophageal varices were found significantly less frequently in 2004 than in 1999. The decrease of frequencies of jaundice and esophageal varices could be explained by several reasons. It was reported that such severe PBC patients who have levels of bilirubin 2+mg/dL seemed not to survive a long time and their 5-year survival rate was 53% in Japan.^2^ Therefore, we tried to compare proportions of patients having levels of bilirubin 2+mg/dL between 1999 and 2004, and could find that the proportion in 1999 was higher than in 2004 (9.0% and 5.9%, respectively). From this result, it is considered that the patients with a high level of bilirubin who often had esophageal varices died within 5 years and the frequency of jaundice in 2004 decreased. It is well known that an elevated bilirubin level is an important prognostic value among the patients with PBC.^3^

Recently, usage of ursodeoxycholic acid (UDCA) is a very common treatment for PBC, and it is known that UDCA lowers the serum level of ALP and γ-GTP, especially among the patients of early stage of PBC.^14^^-^^16^ In our results, levels of γ-GTP and IgM were significantly lower in 2004 than in 1999, although the level of ALP did not decrease. Usage of UDCA is now common in Japan as well as in western countries; thus UDCA might be effective to lower the level of γ-GTP in the present study. The mechanism of effects of UDCA is still unclear, but it is considered that the drug may have cytoprotective and choleretic effects and alters the bile pool by competition for uptake by ileal bile acid receptors.^3^ In the present study, the 95 percentile of the level of total bilirubin was higher in 1999 than in 2004, thus more frequency of patients with high bilirubin level in 1999 was considered a main reason for significant difference between the two years.

The positive rate of AMA was significantly higher among the patients in 1999 than in 2004. Previously, Michieletti et al. described patients with features like PBC in whom serum AMA was negative and antinuclear antibodies were positive,^17^ and they suggested a subgroup termed autoimmune cholangitis. AMA is generally examined by the immunofluorescence method (IF) and/or by enzyme-linked immunosorbent assay (ELISA),^18^^,^^19^ and AMA in the present study was also examined by IF and/or ELISA. Therefore, assessment of the positivity of AMA is thought to be reliable, and it may be possible that AMA negative patients belong to autoimmune cholangitis. We have already reported AMA negative patients with PBC in 1999 among Japanese^20^ who showed a lower level of serum IgM. In the present study, the level of IgM was lower in 2004 than in 1999. However, we cannot immediately conclude that the number of the patients with autoimmune cholangitis is gradually increasing because we could not obtain adequate information about histopathology findings in 2004.

Complicated autoimmune diseases such as Sjögren's syndrome, rheumatoid arthritis, and chronic thyroiditis were found significantly more frequent in 2004 than in 1999. In Western countries it is reported that Sjögren's syndrome, rheumatoid arthritis, and thyroid diseases are found in 20%, 10% to 20%, and 10 to 15% of patients with PBC, respectively.^1^^,^^10^^,^^11^ Our AMA negative patients with PBC in 1999 had higher frequencies of complicated autoimmune diseases than AMA positive patients.^20^ These higher frequencies of complicated autoimmune diseases may also suggest increases of autoimmune cholangitis

The present study has some limitations. Firstly, we could not access all of the data from registered patients with PBC because several prefectures did not provide the data. Moreover, there were many blank spaces in data regarding resident areas of the patients in 1999, and only 2,127 cases were available. Secondly, we could not completely discuss the AMA negative patients with respect to autoimmune cholangitis because we could not obtain the histological information for all of the patients in 2004. Finally, we compared the cross-sectional clinical features of the patients with PBC in different two years, but comparison of the same patients during some periods is more desirable when we want to know the clinical courses of the patients with PBC. Therefore, we are planning to examine corresponding patients at different points in time.

In conclusion, among the patients with PBC in 2004, an increase in median age, and lower levels of laboratory data such as γ-GTP have been found compared to 1999. These results may show an accumulation of patients with better prognosis and the recent medical progress in controlling patients with PBC.
